# The Impact of Myeloperoxidase and Activated Macrophages on Metaphase II Mouse Oocyte Quality

**DOI:** 10.1371/journal.pone.0151160

**Published:** 2016-03-16

**Authors:** Faten Shaeib, Sana N. Khan, Mili Thakur, Hamid-Reza Kohan-Ghadr, Sascha Drewlo, Ghassan M. Saed, Subramaniam Pennathur, Husam M. Abu-Soud

**Affiliations:** 1 Department of Obstetrics and Gynecology, the C.S. Mott Center for Human Growth and Development, Wayne State University School of Medicine, Detroit, MI 48201, United States of America; 2 Division of Nephrology, Department of Internal Medicine, University of Michigan Medical School, Ann Arbor, Michigan, United States of America; 3 Department of Biochemistry and Molecular Biology, Wayne State University School of Medicine, Detroit, MI 48201, United States of America; The Hospital for Sick Children and The University of Toronto, CANADA

## Abstract

Myeloperoxidase (MPO), an abundant heme-containing enzyme present in neutrophils, monocytes, and macrophages, is produced in high levels during inflammation, and associated with poor reproductive outcomes. MPO is known to generate hypochlorous acid (HOCl), a damaging reactive oxygen species (ROS) utilizing hydrogen peroxide (H_2_O_2_) and chloride (Cl^-^). Here we investigate the effect of activated immune cells and MPO on oocyte quality. Mouse metaphase II oocytes were divided into the following groups: 1) Incubation with a catalytic amount of MPO (40 nM) for different incubation periods in the presence of 100 mM Cl^-^ with and without H_2_O_2_ and with and without melatonin (100 μM), at 37°C (n = 648/648 total number of oocytes in each group for oocytes with and without cumulus cells); 2) Co-cultured with activated mouse peritoneal macrophage and neutrophils cells (1.0 x 10^6^ cells/ml) in the absence and presence of melatonin (200 μM), an MPO inhibitor/ROS scavenger, for different incubation periods in HTF media, at 37°C (n = 200/200); 3) Untreated oocytes incubated for 4 hrs as controls (n = 73/64). Oocytes were then fixed, stained and scored based on the microtubule morphology and chromosomal alignment. All treatments were found to negatively affect oocyte quality in a time dependent fashion as compared to controls. In all cases the presence of cumulus cells offered no protection; however significant protection was offered by melatonin. Similar results were obtained with oocytes treated with neutrophils. This work provides a direct link between MPO and decreased oocyte quality. Therefore, strategies to decrease MPO mediated inflammation may influence reproductive outcomes.

## Introduction

There are many challenging questions and issues surrounding poor reproductive outcomes. Many of these problems have come to the forefront of the medical field with greater expectations from medical science. A substantial body of literature has proposed a link between oxidative stress and poor reproductive outcomes [1–3]. Oxidative stress, generated by reactive oxygen species (ROS) overproduction [1, 4] or myeloperoxidase (MPO) activity [5, 6], plays a central role in inflammation that causes these conditions [7, 8]. The deleterious actions of activated macrophages, the major source for ROS and MPO, are secondary to their ability to migrate to any site in the female genital tract and cause their cellular effects at the level of the oocyte [9–13]. Under normal and inflammatory conditions, activated macrophages are found in the cumulus cell mass within the cumulus oocyte complex (COC) [11, 12]. At sites of inflammation, the amount of MPO generated has been reported to reach a concentration of 1–2 mM [14]. High levels of MPO have been found in the collected peritoneal fluid samples of patients with chronic genital diseases [15, 16], polycystic ovarian syndrome (PCOS) [17, 18], advanced stages of endometriosis [16, 19, 20], and pelvic inflammatory disease [16, 21, 22]. Moreover, elevated MPO levels have also been found in the follicular fluid of women with chronic anovulation [23], which correlated to a decline in their fertility.

Myeloperoxidase generates hypochlorous acid (HOCl) through MPO activity in the presence of chloride (Cl^-^) and hydrogen peroxide (H_2_O_2_) [24, 25]. Activated neutrophils generate around 150−425 μM HOCl/hr, while at areas of inflammation, the HOCl level can be reach as high as 5 mM [26, 27]. Under these conditions, HOCl not only destroys invading pathogens but can also cause damage through its capacity to react with other biomolecules, including aromatic chlorination, aldehyde generation, chloramine formation, and oxidation of thiols [4, 28]. Accumulation of HOCl can also mediate hemoprotein heme destruction and subsequent free iron release and protein aggregation through a feedback mechanism involving MPO deterioration [29]. Both, HOCl and increased iron levels have been involved in several inflammatory conditions such as endometriosis [19, 30]. HOCl is much more powerful oocyte aging accelerant than other ROS through its ability to deteriorate the oocyte microtubule morphology (MT) and chromosomal alignment (CH), which are markers of oocyte quality [4]. Although MPO and HOCl are found in large amounts during inflammation contributing to poor reproductive outcomes, little is known about the exact mechanisms through which MPO affects oocyte quality.

Recently, utilizing HPLC and amperometric integrated H_2_O_2_-selective electrode, our group demonstrated real time in vivo measurements of intracellular H_2_O_2_ and its ability to diffuse outside the oocyte to activate extracellular MPO generating HOCl [31]. The ability of this investigation to provide a precise measurement of in situ H_2_O_2_ was secondary to limiting reactivity with nearby biological processes and minimizing loss caused by diffusion. Thus, it could be demonstrated through the use of catalase that the measurements were that of H_2_O_2_ and not an unknown substance in our system [31]. Our results showed that the diffused H_2_O_2_ triggered MPO chlorinating activity, which in turn facilitated oocyte quality deterioration, which was shown to be preventable if oocytes were pre-treated with melatonin. Melatonin, a known pineal hormone involved in the regulation of circadian rhythms [32, 33] has identified as a potent inhibitor of MPO chlorination activity and a potent scavenger of its final product, HOCl [34–36]. The beneficial effect of melatonin on oocyte quality and fertilization has been previously described [36–39].

The current study demonstrates that MPO has a detrimental effect on oocyte quality through its chlorination activation, and defines the link between MPO (purified and naturally secreted from macrophages and neutrophils) and oocyte quality (MT and CH) deterioration, a mechanism that can be prevented by using melatonin. These results may help in designing treatment plans for assisted reproductive technologies for patients with inflammatory conditions.

## Materials and Methods

### Materials

Hydrogen peroxide, melatonin, Human tubular fluid (HTF) media were purchased from life technology, anti-α tubulin antibody, and Alexa Fluor^®^ 488-AffiniPure Goat Anti-Mouse IgG (H+L) were purchased Jackson ImmunoResearch. Propidium iodide (PI), 1% bovine serum albumin (BSA), 0.1% M glycine, 0.1% Triton X-100, sodium nitrite, and trypan blue, lipopolysaccharide (LPS) were purchased from Sigma Aldrich (St. Louis, MO, USA). Normal goat serum 2% was purchased from Invitrogen and powdered milk, 0.2%, was obtained from the grocery store. Peritoneal macrophage cells (non-stimulated, adherent, and non-dividing) derived from female C57BL/6 mice were obtained from Astarte Biologics, LLC (Bothell, WA) (1 x 10^6^ /1ml), macrophage cell media and other supplements were also obtained from ScienCell Research Laboratories, Inc. (Carlsbad, CA). The macrophage media (DMEM) with its supplemented materials and 10% Fetal Bovine Serum (FBS) were obtained from Science Cell Research Laboratories (Carlsbad, CA). Peritoneal neutrophil cells (non-stimulated, adherent, and non-dividing) derived from female C57BL/6 mice were obtained from Astarte Biologics, LLC (Bothell, WA) (1 x 10^6^ /1ml), neutrophil cell media and other supplements were also obtained from ScienCell Research Laboratories, Inc. (Carlsbad, CA).

Other chemicals and reagents were of the highest purity grades available and obtained from Sigma Aldrich.

### Methods

#### Myeloperoxidase Purification

Myeloperoxidase was purified initially from detergent extracts of human leukocytes by sequential lectin affinity and gel-filtration chromatography [40–42]. Trace levels of eosinophil peroxidase that may be contaminating were then removed by passage over a sulfopropyl Sephadex column [41]. Purity of isolated MPO was established by demonstrating a Reinheitzal (RZ) value of 0.85 (A430/A280), SDS–PAGE analysis with Coomassie blue staining, and gel tetra- methylbenzidineperoxidase staining to the absence of eosinophil peroxidase activity. Enzyme concentration was determined spectrophotometrically utilizing extinction coefficients of 89,000 M^−1^ cm^−1^/heme of MPO [43].

#### Hydrogen peroxide solution

The H_2_O_2_ solutions were prepared fresh in phosphate buffer (PH 7.4), after which the concentration of the working solutions was determined spectrophotometrically (extinction coefficient of 43.6 M^-^1 cm^-^1 at 240 nm) [44, 45]. During the preparation process, all the solutions were kept on ice to minimize decomposition.

Melatonin solution: A stock solution of melatonin was dissolved in dimethylformamide (DMF) and diluted to the required concentration with phosphate buffer (pH = 7.4). The final concentration of DMF in all melatonin solutions was less than 1% and did not interfere with MPO activity or have any effect on oocyte quality [46].

#### Oocyte preparation

Metaphase II mouse oocytes (with and without cumulus cells) were obtained from a B6C3F1 mouse crossed with a B6D2F1 mouse in cryopreserved straws using ethylene glycol-based slow freeze cryopreservation protocol (Embryotech Lab). Oocytes at this stage were used as they are arrested in a mature stage just prior to fertilization and demonstrate a dense array of filaments with bundles forming the meiotic spindle as the scaffold for segregation of genetic material [2, 47]. Metaphase II (MII) oocytes are known to be exposed to some ROS during ovulation [4, 48, 49]. The major functional parameters used to assess oocyte quality at this stage are spindle microtubule morphology (MT), chromosomal alignment (CH) and organization of the cumulus oocyte complex (COC) as it has been established that they can be affected by changes in the oocyte microenvironment such as increased ROS. Furthermore, we chose to use frozen-thawed oocytes instead of fresh as both our group and others have performed many experiments on both and found that treatment of fresh and frozen oocytes with ROS had yielded similar and reproducible results [4, 50–54]. Institutional Review Board approval was not required, as the oocytes were obtained from Embryotech. Oocytes were transferred from straws to phosphate-buffered saline (Dulbeco’s PBS) and washed to remove excess cryoprotectant for 5 minutes. Oocytes were then transferred to HTF media and incubated at 37°C and 5% CO_2_ for 60 minutes to allow repolymerization of spindles. The oocytes were then screened for the presence of polar bodies confirming their metaphase II stage [5]. Ten to twenty oocytes from each group were discarded as they were found to be immature or displayed disrupted zona pellucida (ZP).

### Purified myeloperoxidase treatment on oocytes (with and without cumulus cells)

Using the same processes for oocytes handling as mentioned in the previous section, metaphase II mouse oocytes with (n = 648) and without cumulus cells (n = 648) were divided into the following groups, which were performed in triplicate. Oocytes were divided into groups with and without cumulus cells to study the protective effect of cumulus cells.

Group 1 (n = 120 cumulus/n = 120 without cumulus): oocytes incubated with fixed concentration of MPO (40 nM) at different incubation periods (3, 6, 12, and 24 hrs); group 2 (n = 120 cumulus/n = 120 without cumulus): oocytes incubated with fixed concentration of MPO (40 nM) + 20 μM H_2_O_2_ at different incubation periods (3, 6, 12, and 24 hrs); group 3 (n = 108 cumulus/n = 108 noncumulus): oocytes incubated with fixed concentration of MPO (40 nM) at different incubation periods (3, 6, 12, and 24 hrs) preincubated with melatonin (100 μM); group 4 (n = 108 cumulus/n = 108 without cumulus): oocytes incubated with fixed concentration of MPO (40 nM) + H_2_O_2_ (20 μM) at different incubation periods (3, 6, 12, and 24 hrs) on oocytes preincubated with melatonin (100 μM); group 5 (n = 120 cumulus/n = 120 without cumulus): Untreated oocytes were used as a control; and group 6 (n = 72 cumulus/n = 72 without cumulus): oocytes with melatonin (100 μM) alone for 24 a hr incubation period. In both groups melatonin was added to media immediately prior to addition of MPO or other compounds. All oocytes were fixed at the time points (3, 6, 12, and 24 hrs) and evaluated for alteration of the following: MT structure and CH alignment. All experiments were carried out in HTF media containing 100 mM Cl^-^, which is similar to the physiological oviduct Cl^-^ concentration [55]. All cell transfers were performed using 200-mm micropipette tips (ORIGIO, Cooper Surgical).

### Macrophage cells co-cultured with oocytes (with and without cumulus cells)

We followed the protocol of macrophage cell co-culture as described by Honda et al (1994) [56] with some modifications. The 1 ml vials containing macrophage cells (1.0 x 10^6^ cells/vial) were thawed at 37°C then centrifuged at 1800 rpm at 4°C for 5 min then the cryopreservative solution was removed and replaced with the macrophage media, mixed, and 1 μl of the media containing macrophage cells was used to test the cell viability using Trypan blue dye exclusion assay. Cells were recounted before utilization and were placed into 30 mm dishes (Falcon). The macrophage concentration per dish was chosen secondary to previous data which showed a significant reduction in fertilization rate in the co-culture group as compared to control [56]. Macrophage cells were stimulated with lipopolysaccharide (LPS) (10 ng/ml) for maximal MPO secretion [57, 58]. Cells were allowed to rest for 16 hr at 5% CO_2_, 37°C to allow the cells to adhere to the base of culture dishes. The following day, the macrophage media was removed, washed with PBS twice, and then with HTF twice to remove the unadherent cells and replaced with HTF media. Cells were then reincubated at 5% CO_2_, 37°C to be ready for co-cultured with the oocytes.

In the triplicate experiment, the oocytes (with n = 200 and without cumulus cells n = 200, total) were divided into the following groups: group 1: oocytes with and without cumulus cells incubated with stimulated macrophage cells for 1, 2, 3, and 4 hrs; group 2: oocytes with and without cumulus cells incubated with stimulated macrophage cells preincubated with a higher concentration of melatonin (200 μM) to demonstrate the protective effect against higher amounts of MPO for 1, 2, 3, and 4 hrs; group 3: oocytes with and without cumulus cells incubated with 100 μM melatonin alone for 4 hrs; group 4: control oocytes receiving no treatment and incubated for 4 hrs. All groups were incubated in HTF for 4 hrs, 37°C, 5% CO_2_. We chose 4 hrs of incubation as previous studies stated that 4–6 hrs is the time for optimal fertilization [59, 60]. The doses of melatonin and HOCl were selected on the basis of our preliminary results and our previous studies [35, 36]. All oocytes were fixed at the time points (1, 2, 3, and 4 hrs) and evaluated for alteration of the following: MT structure and CH alignment.

### Neutrophil cells co-cultured with oocytes (with and without cumulus cells)

The 1 ml vials containing neutrophil cells (1.0 x 10^6^ cells/vial) were thawed at 37°C then centrifuged at 1800 rpm at 4°C for 5 min then the cryopreservative solution was removed and replaced with the neutrophil media, mixed, and 1 μl of the media containing neutrophil cells was used to test the cell viability using Trypan blue dye exclusion assay. Cells were recounted before utilization and were placed into 30 mm dishes (Falcon). The neutrophil concentration per dish was chosen secondary to previous data which showed a significant reduction in fertilization rate in co-culture group as compared to control [56]. Neutrophils were stimulated with lipopolysaccharide (LPS) (10 ng/ml) for maximal MPO secretion [57, 58]. Cells were allowed to rest for 16 hr at 5% CO_2_, 37°C to allow the cells to adhere to the base of culture dishes. The following day, the neutrophil media was removed, washed with PBS twice, and then with HTF twice to remove the unadherent cells and replaced with HTF media. Cells were then reincubated at 5% CO_2_, 37°C to be ready for co-cultured with the oocytes.

In the experiment, the oocytes (with n = 100 and without cumulus cells n = 100, total) were divided into the following groups: group 1: oocytes with and without cumulus cells incubated with stimulated neutrophil cells for 1, 2, 3, and 4 hrs; group 2: control oocytes receiving no treatment and incubated for 4 hrs. All groups were incubated in HTF for 4 hrs, 37°C, 5% CO_2_. We chose 4 hrs of incubation as previous studies stated that 4–6 hrs is the time for optimal fertilization [59, 60]. All oocytes were fixed at the time points (1, 2, 3, and 4 hrs) and evaluated for alteration of the following: MT structure and CH alignment.

### Immunofluorescence staining and fluorescence microscopy

Oocytes were fixed in a solution prepared from 2% formaldehyde and 0.2% Triton X-100 for 30 minutes and then treated with blocking solution (PBS, 0.2% Powdered Milk, 2% Normal Goat Serum, 1% BSA, 0.1 M Glycine and 0.1% Triton X-100) for 1 hr followed by PBS washing for 3 minutes. The oocytes were then subjected to indirect immunostaining using mouse primary anti-α tubulin antibody against the MT (1:100, overnight) and secondary Alexa Fluor^®^ 488-AffiniPure Goat Anti-Mouse IgG (H+L) (1:50, 1 h). The secondary antibody used was specifically able to bind to our primary antibody. As a control, we incubated the oocytes with secondary antibody for the same incubation time used for our experiments and observed no spindle signals in the oocytes. The chromosomes were stained using PI for 10 min. Stained oocytes were loaded into anti-fade agent on slides with two etched rings. Images were obtained utilizing both immunofluorescence and confocal microscopy.

### Confocal microscopy, assessment of microtubules and chromosomal alignment

Confocal microscopy, assessment of microtubules morphology and chromosomal alignment slides were examined with the Zeiss LSM 510 META NLO (Zeiss LSM 510 META) microscope using PI (red) and Alexa Fluor^®^ 488 (green) fluorescent filters with excitation and emission wavelengths of 470 and 525 nm, and 495 and 519 nm, respectively. Oocytes were localized using a 10x magnification lens and spindle alterations assessed using 100x oil immersion lens. The MT was stained fluorescent green, which was distinct from the fluorescent red staining of chromosomes. The alterations in the MT and CH were compared with controls and scored by three different observers blinded to treatment groups based on a previously published scoring system using comprehensive evaluation of the individual optical sections and the 3-D reconstructed images [36, 61]. Scores of 1–4 were assigned for both MT and CH alterations, with scores 1 and 2 combined for good outcomes meaning microtubules were organized in a barrel-shaped with slightly pointed poles formed by organized microtubules crosswise from one pole to and chromosomes were normally arranged in a compact metaphase plate at the equator of the spindle. Scores of 3 and 4 signified poor outcomes and consisted of spindle length reduction, disorganization and/or complete spindle absence, and chromosome dispersion or aberrant condensation appearance.

### Detection of ROS generation in oocytes after exposure to MPO

The oocytes without cumulus cells (n = 20) were incubated with MPO (40 nM) or without (control, n = 20) for 24 hours. Generation of ROS was evaluated using the Cellular Reactive Oxygen Species Detection Assay Kit Abcam (ab186029 (Cambridge, UK)) as instructed by manufacturer. After fixation by 2% formaldehyde and permeabilization with 0.2% Triton X-100 for 30 minutes, nuclei were stained with DAPI. Images of ROS-mediated deep red fluorescence were taken using a Nikon Eclipse 90i epifluorescence microscope. The fluorescence intensity of each oocyte image was measured from quantification of mean pixel intensities using NIS-Element (Nikon, Shinagawa-Ku, Tokyo, Japan). The overall fluorescence unit for MPO treatment group of oocytes was calculated relatively to control group and represented as normalized relative fluorescent unit (RFU). The Student's *t* -test was performed on the RFU to test if the difference between control and MPO treatment groups is statistically significant (p<0.05).

#### Statistical analysis

Statistical analyses were performed using SPSS version 21.0 (SPSS Inc., Chicago, IL, USA). One-way ANOVA analyses were performed to compare the percentage of oocytes with poor outcomes (scores 3 and 4) for MT and CH between controls with various time intervals and each treatment group applied to cumulus and non-cumulus oocytes. The same ANOVA procedures were also performed to investigate the effects of different treatments on cumulus and non-cumulus oocytes at different time intervals. Pair wise comparisons made using Tukey’s post hoc test following significant ANOVA tests, which defined as P<0.05. Independent t tests were conducted to compare the cumulus and non-cumulus oocytes for each treatment and time interval combination.

## Results

### Effect of purified MPO/melatonin on MT and CH of metaphase II oocytes with and without cumulus cells

**Fig 1:**
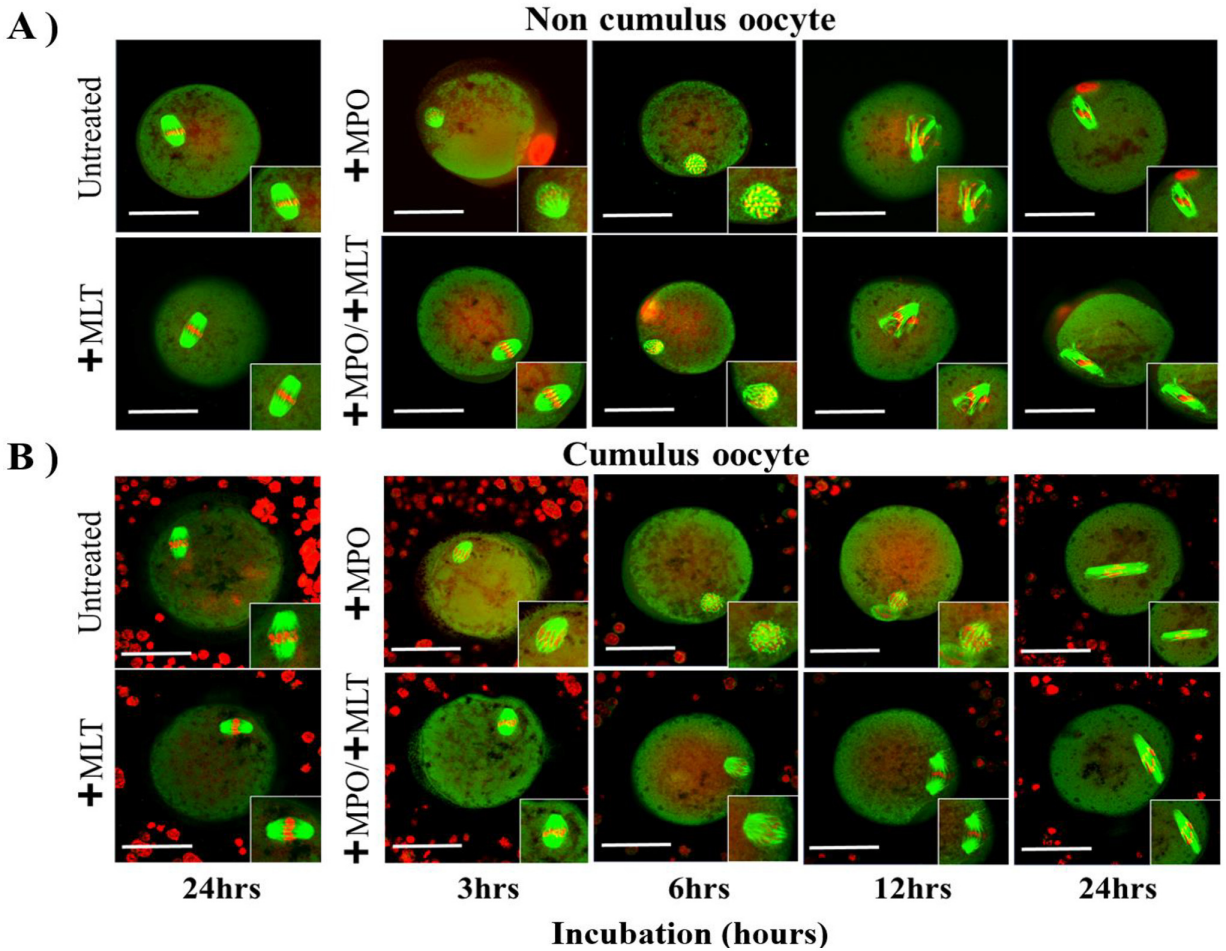
Images of oocytes microtubule morphology (MT) and chromosomal alignment (CH) obtained using Confocal Zeiss LSM 510 META NLO microscope. A) Oocytes without cumulus cells showed: -Upper Panel: Detrimental time dependent effect of MPO (40 nM) on MT and CH. -Lower Panel: MLT supplementation (100 μM) showed normal MT and CH in the presence MPO at 3 hrs of incubation similar to controls and alterations in MT and CH by increasing the incubation time. B) Oocytes with cumulus cells showed similar observations as in A for MPO effect in the absence and presence of MLT. Collectively, cumulus cells failed to offer significant protection against MPO catalytic activity. Scale bars: 50μm. Results depict observations made after three experiments.

To test whether MPO activation through the extra-oocyte diffusion of H_2_O_2_ could deteriorate the mouse metaphase II oocyte quality, we investigated the time dependent effect of MPO, in the absence and presence of melatonin, a potent MPO inhibitor, on oocyte MT and CH in the absence and presence of cumulus cells. Incubation of the oocytes with MPO alone (40 nM) showed significant deterioration in oocytes quality in a time depended manner as judged by alterations in the MT and CH in the treatment groups and cumulus cells show no protection (Fig 1A and 1B-upper Panels). Whereas the presence of 100 μM melatonin showed significant protective effect on MT and CH when incubated with MPO for shorter periods (3 hrs) and this protection was lost at longer time of incubation (Fig 1A and 1B). Pre-supplementing the oocyte medium with more melatonin concentrations (400 μM), showed protection in the oocyte quality up to 6 hrs of incubation with MPO (data not shown). Furthermore, at the 6 hr incubation period in oocytes pretreated with melatonin, a significant protective effect was noted in the cumulus compared to noncumulus group. This indicates that in the 6 hr time period melatonin was able to support cumulus cell antioxidant machinery against HOCl assault, which manifested as preserved oocyte quality. These results concluded that preservation of normal MT and CH under MPO activity could be achieved by using the antioxidant and MPO inhibitor, melatonin.

**Fig 2:**
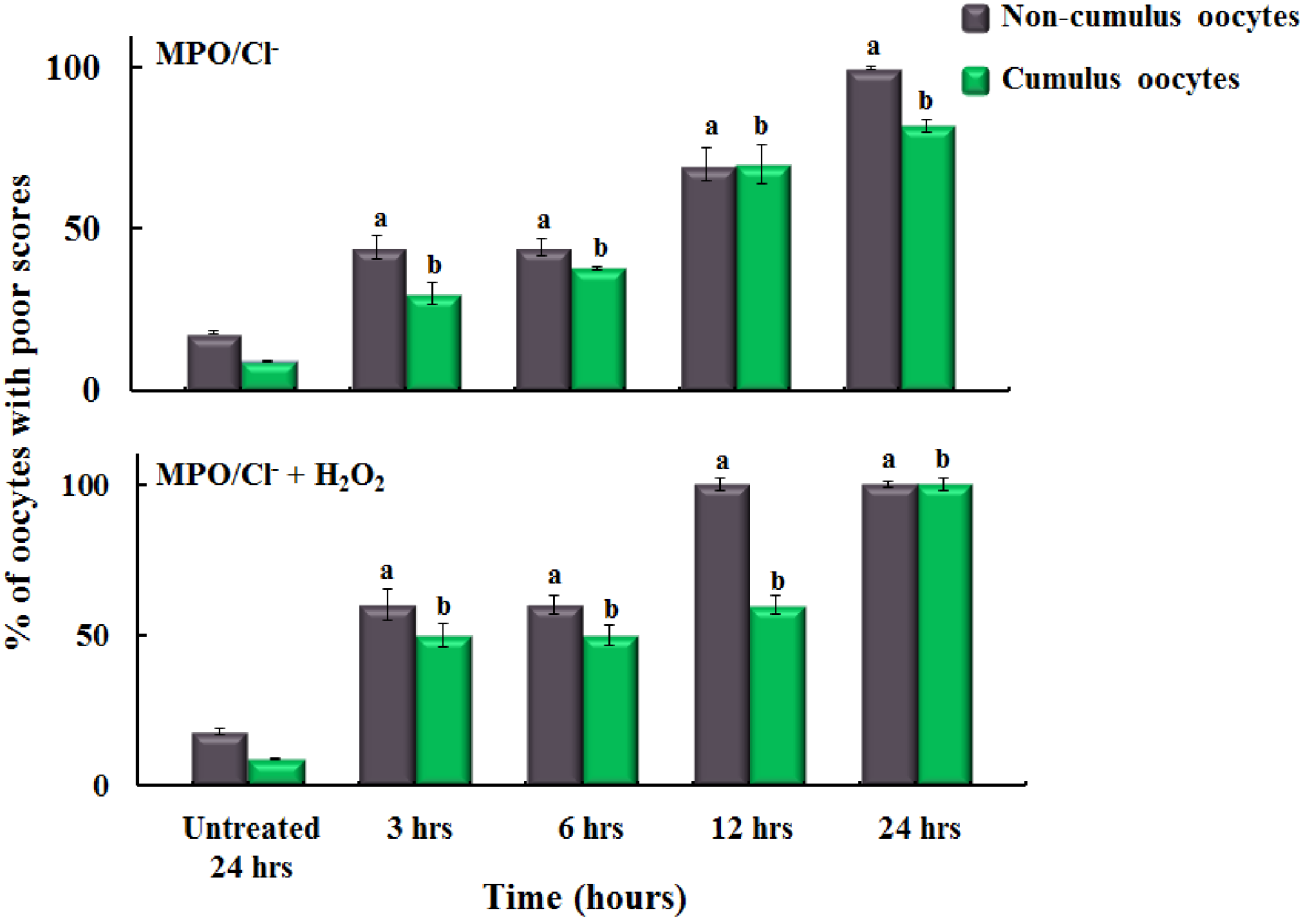
The effect of MPO/HOCl system on oocytes quality in the presence and absence of cumulus cells. Both panels show triplicate experiments of the percentage of oocytes with cumulus (n = 648) (green bars) versus those without (n = 648) (gray bars) with poor microtubule morphology (MT) scores observed in the untreated oocytes compared to oocytes treated with a fixed catalytic MPO concentration (40 nM) without addition of H_2_O_2_ (20 μM) (upper panel) and after addition of H_2_O_2_ (20 μM) (lower panel). All oocytes were incubated to different times of incubation (3, 6, 12 and 24 hrs) followed by indirect immunofluorescence staining method to observe MT and CH. Human tubulin fluid (HTF) media contains similar chloride (Cl^-^) concentration (~100 mM) to the oviduct fluid. There is a time dependent effect of MPO activity on oocytes quality in the presence and absence of cumulus cells (p < 0.05). Cumulus cells fail to protect MT against damage from MPO activity (p > 0.05). Similar results were observed for the chromosomes alignment (CH). One-way ANOVA and independent t-test using SPSS 22.0 used to analyzed the results as following: (a) P < 0.05 non-cumulus oocytes as compared to control. (b) P < 0.05 cumulus oocytes as compared to control. The standard error for each point was estimated to be less than 10%. H_2_O_2_ addition on the oocytes in the lower panel showed no significant difference compared to oocytes in the upper panel.

**Fig 3:**
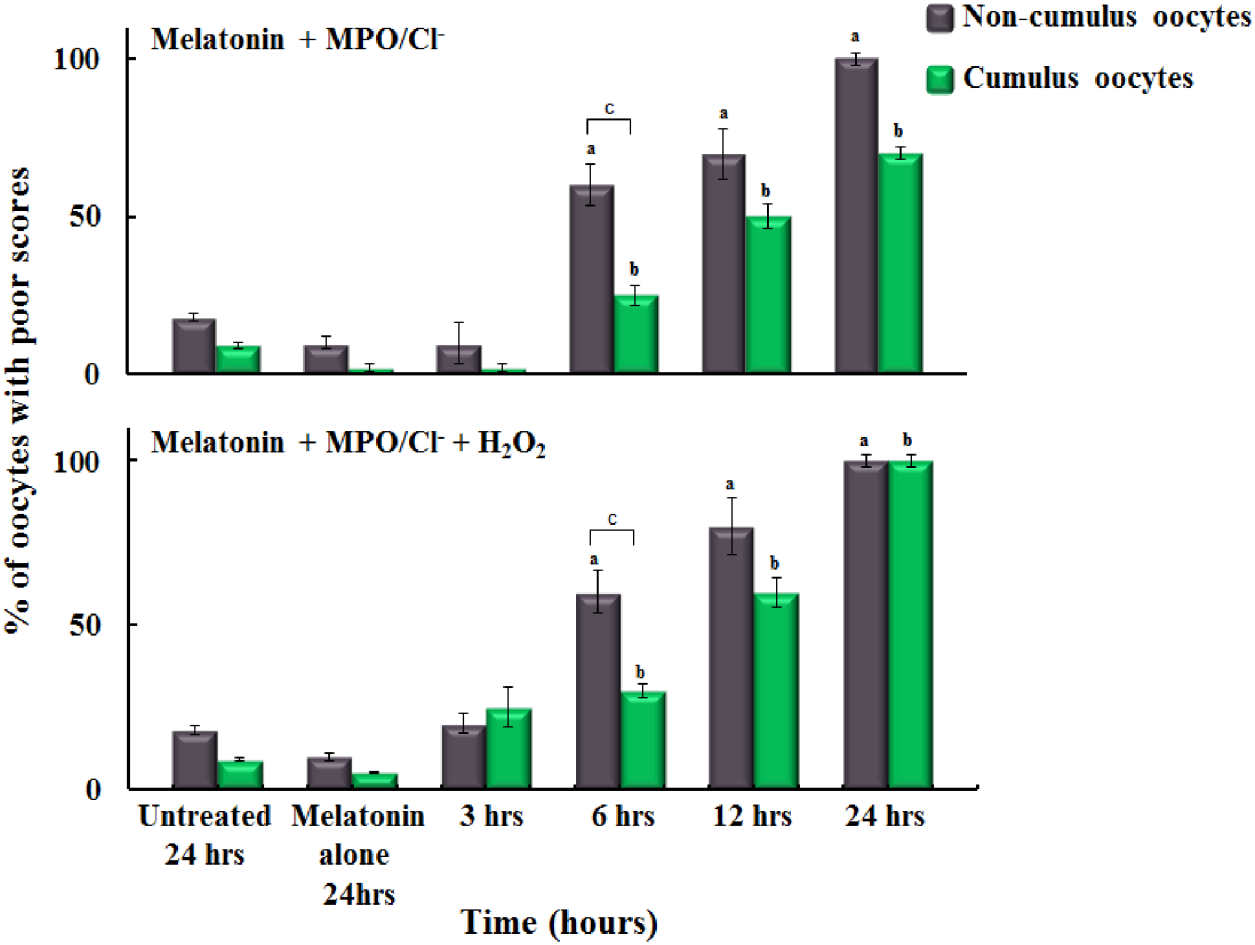
The protective effect of melatonin against MPO/HOCl system on oocytes quality in the presence and absence of cumulus cells. Both panels show triplicate experiments of the percentage of oocytes with cumulus (n = 648) (green bars) versus those without (n = 648) (gray bars) with poor microtubule morphology (MT) scores observed in the untreated oocytes compared to oocytes treated with a fixed catalytic MPO concentration (40 nM) without addition of H_2_O_2_ (20 μM) (upper panel) and after addition of H_2_O_2_ (20 μM) (lower panel) after pre-supplement the oocytes media with melatonin (100 μM). All oocytes were incubated to different times of incubation (3, 6, 12 and 24 hrs) followed by indirect immunofluorescence staining method to observe MT and CH. Human tubulin fluid (HTF) media contains similar chloride (Cl^-^) concentration (~100 mM) to the oviduct fluid. Melatonin showed a significant protection against MPO activity at 3 hrs incubation (p < 0.05) as it works as direct HOCl scavenger and MPO inhibitor. The poor scores in MT showed by increasing the time of incubation caused as melatonin have been consumed. Cumulus cells fail to protect MT against damage from MPO activity (p > 0.05). H_2_O_2_ addition on the oocytes in the lower panel showed no significant difference compared to oocytes in the upper panel. Similar results were observed for the chromosomes alignment (CH). One-way ANOVA and independent t-test using SPSS 22.0 used to analyzed the results as following: (a) P < 0.05 non-cumulus oocytes as compared to control. (b) P < 0.05 cumulus oocytes as compared to control. (c) P < 0.05 between cumulus and non-cumulus oocytes. The standard error for each point was estimated to be less than 10%.

For comparison, the effect of MPO, in the absence and presence of melatonin, on MT and CH were quantitated based on our well-established 1–4 scoring system (see method section for more details) and the percentages of poor scores were plotted as a function of time (Figs 2 and 3). Fig 2 upper Panel showed the time dependent increase in the percentage of poor scores for MT and CH for oocyte with and without cumulus cells incubated with MPO. In the absence of cumulus cells, 3 hr incubation with MPO showed 44.4% poor scoring and stayed almost the same at 6 hr incubation and increased to 70%, and 100% at 12 and 24 hrs, respectively, compared to control group 18%. Similar results were observed for oocytes MT and CH with cumulus cells incubated with the same amount of MPO for the same incubation periods (30%, 38%, 70%, and 82% poor scores, respectively, compared to control groups 9.0%).

Fig 3 showed the protective effect of melatonin on oocyte quality against MPO treatment. Oocytes without cumulus cells incubated with MPO/melatonin showed a significant decrease in the percentages of poor scores MT and CH at 3 hr (7%) of incubation compared to longer incubation periods 6, 12 and 24 hrs (57%, 70%, and 100%, respectively) (p<0.001) (Fig 3 upper panel/gray bars). Whereas, the poor scores for cumulus oocytes incubated for the 3, 6, 12, and 24 hrs with melatonin/MPO were approximately 1.3%, 23.3%, 50%, and 70%, respectively as compared with the control group score average of 1.0% (Fig 3 upper Panel/green bars). In control experiments, oocytes incubated with melatonin alone for 24 hr showed poor scores of ~10.0% similar to untreated oocytes. Cumulus cells showed signs of protection against MPO in the presence of melatonin at 6 hrs of incubation (p<0.05).

**Fig 4:**
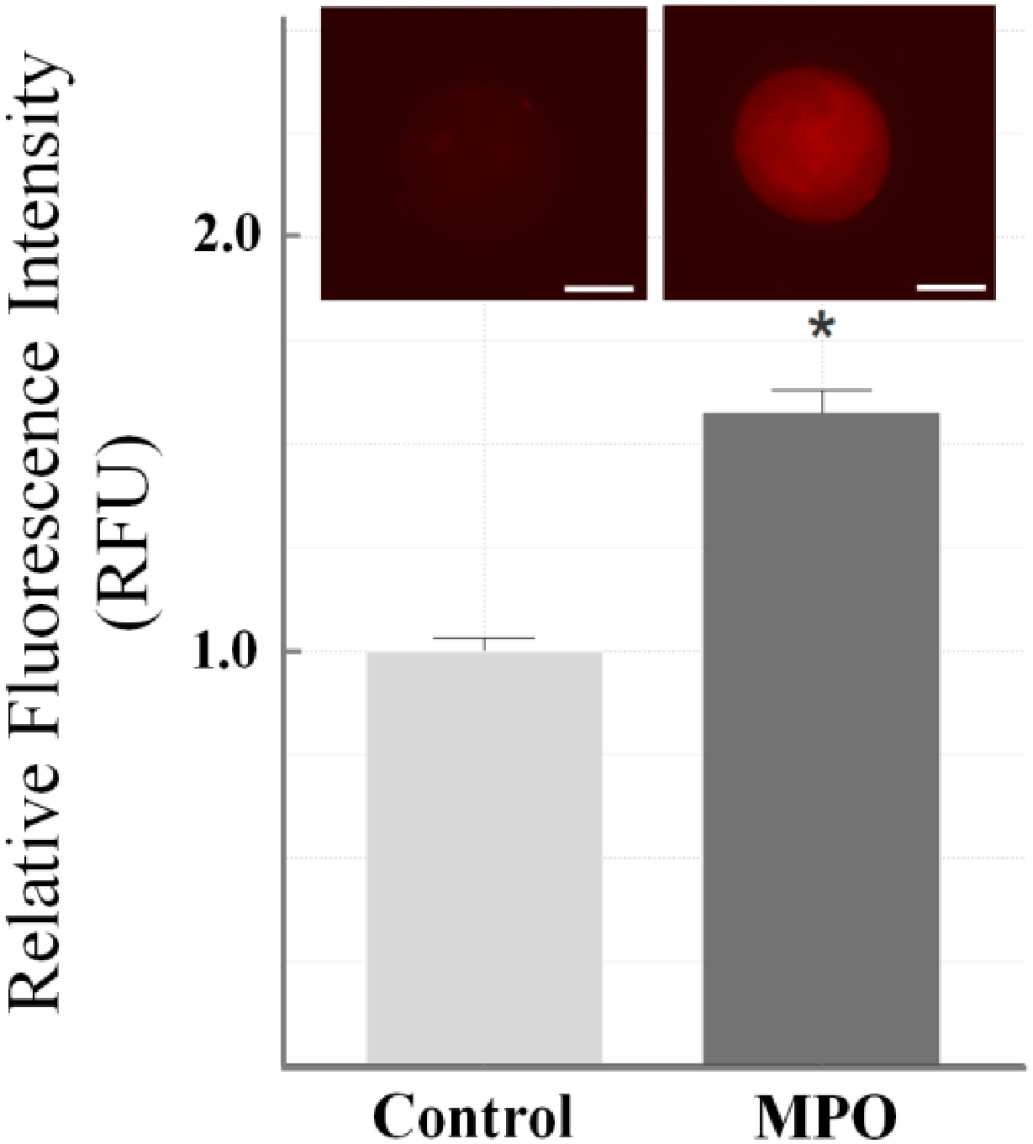
Reactive oxygen species generation in oocytes exposed to MPO. Oocytes without cumulus cells were exposed to MPO (40nM) for 24 hours incubation to determine intracellular ROS generation. The fluorescence intensity was estimated as relative fluorescence unit (RFU) and plotted as a bar graph to present the fold changes in ROS production upon MPO treatment relative to untreated control oocytes. Error bars indicate ± relative standard error of mean (SEM). * p<0.05 vs. controls. Inset presents representative images of control and oocyte exposed to MPO for 24 hours. Scale bars: 50 μm. Images shown are from a typical experiment performed at least three times.

To determine whether MPO activation is the major cause for oocyte quality deterioration, we repeated the same experiments in the presence of exogenously added H_2_O_2_ (20 μM) to the oocyte media immediately after MPO addition and incubated the oocytes for the same incubation times (3, 6, 12 and 24 hrs) in the absence and presence of 100 μM melatonin (Figs 2 and 3-lower Panels). Our results showed that in the absence of cumulus cells, the poor scores for MT and CH for 3, 6, 12 and 24 hrs of incubation periods with MPO/H_2_O_2_ alone were 60%, 60%, 100% and 100% respectively (Fig 2-lower Panel/gray bars). In the presence of cumulus cells, the percentage of poor scores for 3, 6, 12, and 24 hrs of incubation periods with MPO/H_2_O_2_ alone were 50%, 50%, 60%, and 100% respectively (Fig 2-lower Panel/green bars). Results in this section mirrored those of the above experiment (Fig 2, upper Panel) in which H_2_O_2_ supplementation had no additional effect on oocyte quality (p>0.05), and again cumulus cells did not appear to provide protection to the oocyte (p>0.05). The poor scores of non-cumulus oocytes (for MT and CH) incubated with melatonin/MPO/H_2_O_2_ for 3, 6, 12, and 24 hr incubation were 20%, 63.3%, 73.3%, and 100% respectively (Fig 3 lower Panel/gray bars). Under these circumstances, melatonin also showed protection at 3 hrs of incubation, but not in the other incubation periods. In the presence of cumulus cells, the poor scores for oocytes MT and CH incubated with the same treatments for the same incubation periods were 25%, 60%, 60%, and 100% respectively (Fig 3 lower Panel/green bars). Results in this section mirrored results without addition of H_2_O_2_, in that the presence of melatonin protected against MPO with H_2_O_2_ in the 3 hr group (p<0.001); but not in the 6, 12, or 24 hrs groups (p>0.05). Cumulus cells did not showed a protective effect after 6 hrs of incubation in the presence of melatonin and MPO with H_2_O_2_ (p>0.05). Collectively, inhibiting MPO activity by using melatonin preserved the quality of the oocytes, thus MPO activity was the major cause of poor oocyte quality. To better understand the mechanism through which MPO affects oocyte quality, ROS generation was monitored. Oocytes were incubated with MPO for 24 hrs, where the maximum negative effect of MPO was observed, and significant increase in ROS generation was noted as compared to control (Fig 4). The inset of Fig 4 shows the treatment oocyte saturated with red color indicating ROS generation as compared to the control oocyte demonstrating less color, this effect was quantitated in terms of relative fluorescence units (RFU) and the difference showed statistical significance (p<0.05). Collectively, these results demonstrate that MPO deteriorates oocyte quality in a time dependent manner through an ROS mediated mechanism and melatonin displays some protective ability against these insults.

### Effect of stimulated macrophages and neutrophils on MT and CH of metaphase II oocytes without and with cumulus cells

**Fig 5:**
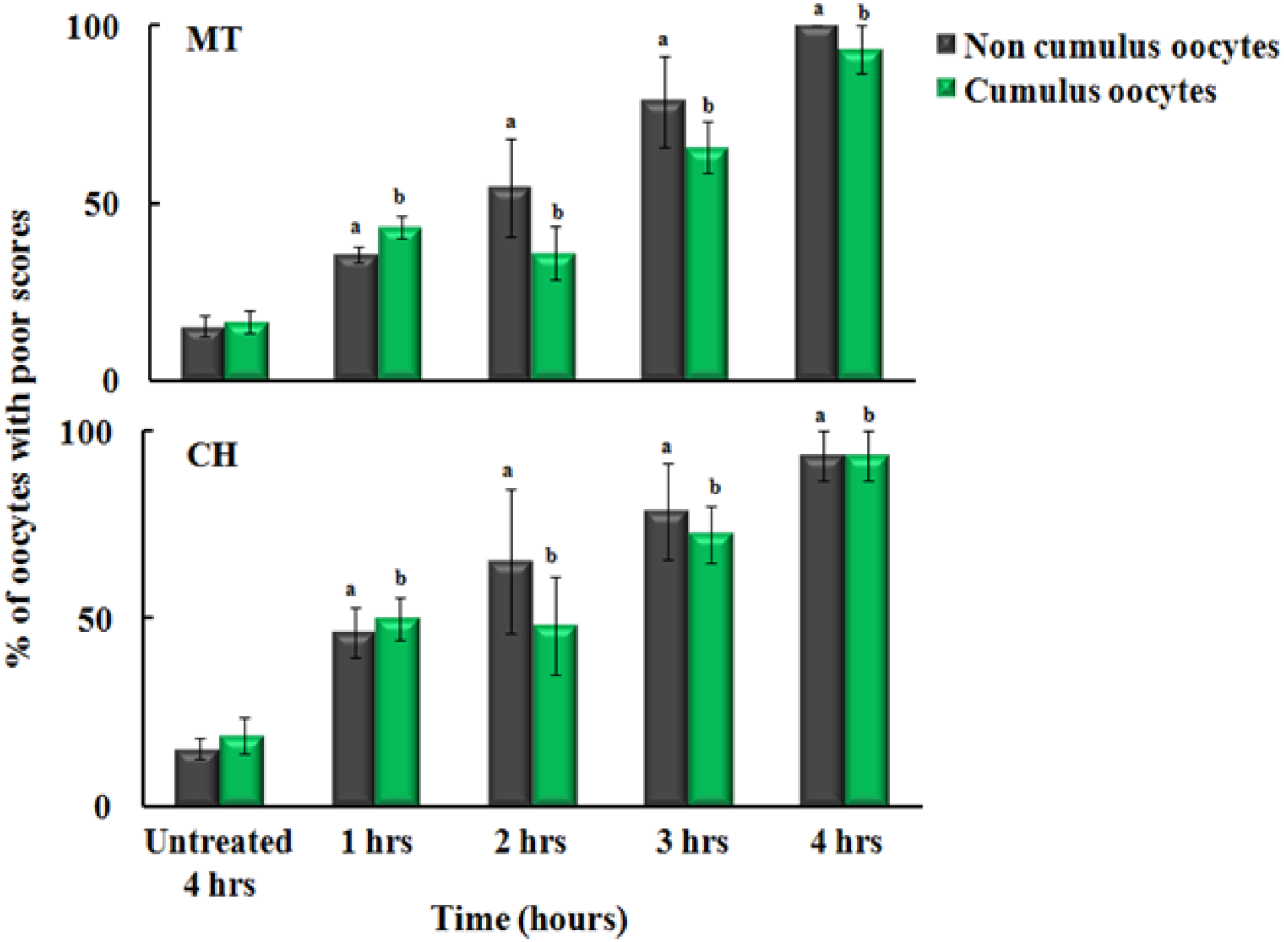
The direct effect of stimulated macrophages on oocyte quality, microtubule morphology (MT) and chromosomal alignment (CH), in the absence (gray bars) and presence (green bars) of cumulus cells. There was a significant time dependent effect of stimulated macrophages on MT and CH (p<0.05). Cumulus cells did not offer significant protection against macrophages activity (p>0.05). The experiments were conducted with three replications and the error bars represent the standard error of the mean.

**Fig 6:**
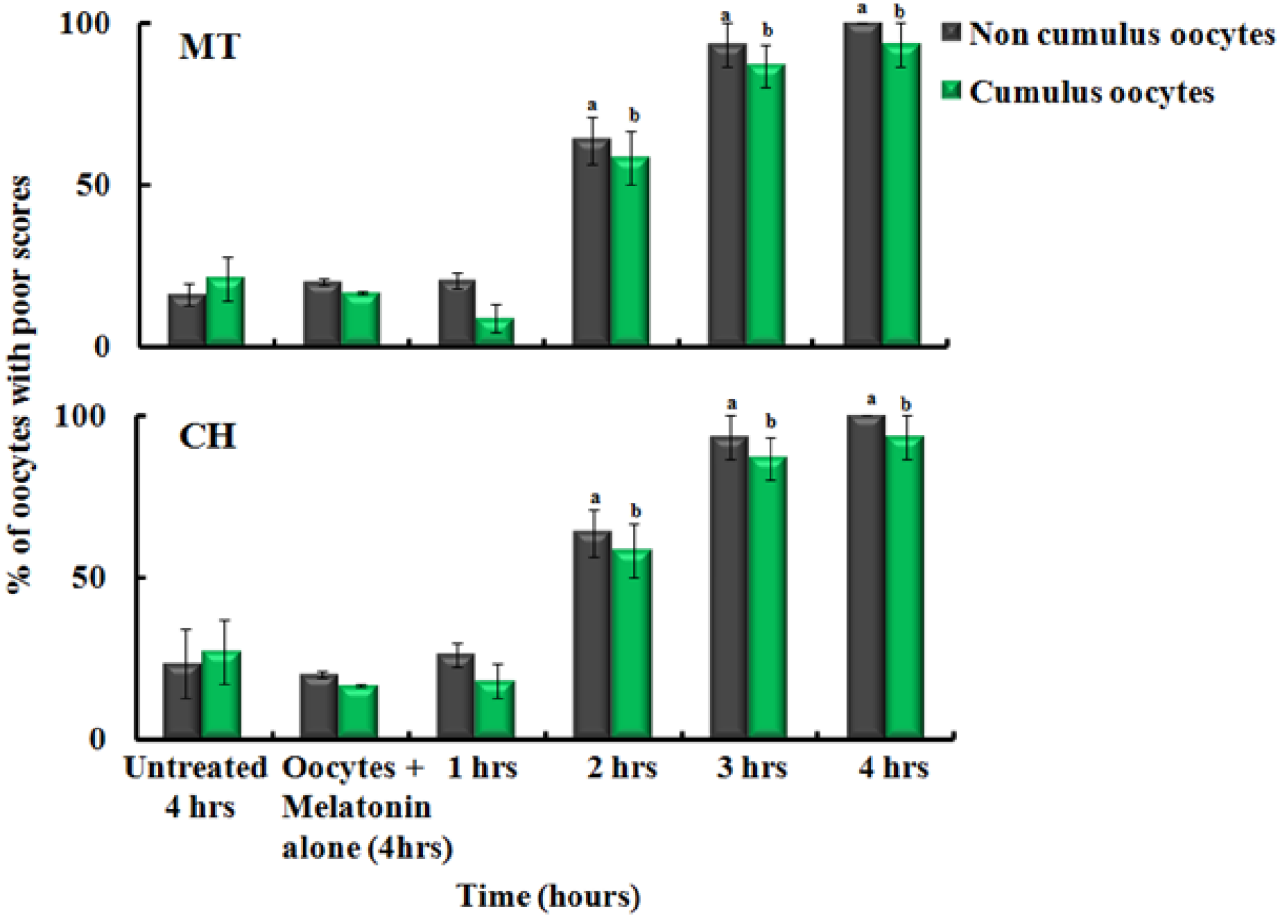
The protective effect of melatonin (MLT) against stimulated macrophages activity on oocytes quality (MT and CH) in the absence (gray bars) and presence (green bars) of cumulus cells. The presence of melatonin (200 μM) showed significant protection for MT and CH at 1 hr incubation (p<0.05). In general, cumulus cells did not offer significant protection against macrophages activity (p>0.05). The experiments were conducted with three replications and the error bars represent the standard error of the mean.

Since purified MPO activity was responsible for oocyte quality deterioration, then we test whether the exposure to stimulated macrophages could mediate deterioration of the oocytes quality through a mechanism that involves the MPO catalysis. To test this hypothesis, we co-cultured the oocytes with stimulated macrophages as function of time, in the presence and absence of melatonin (200 μM). As showed in Fig 5, in the absence of cumulus cells, the poor scores for the oocytes incubated with stimulated macrophage for 1, 2, 3 and 4 hrs were approximately 35.5%, 54.4%, 78.5% and 100% for MT and CH (Fig 5). In the presence of cumulus cells, the average poor scores for the oocytes incubated with stimulated macrophage cells for 1, 2, 3 and 4 hrs was approximately 43.3%, 36.1%, 65.7% and 93.3% for MT and CH (Fig 5). Overall, as showed in Fig 2, increasing the incubation time increased significantly the poor scores for MT and CH (p<0.05). Fig 6 demonstrated the power of melatonin to inhibit the activity of naturally MPO secreted from stimulated macrophages. The poor scores for noncumulus oocytes MT and CH incubated with melatonin/stimulated macrophage cells for 1, 2, 3 and 4 hrs were approximately 20.5%, 63.8%, 93.3% and 100% (Fig 6). While, in the presence of cumulus cells, the poor scores for MT and CH for 1, 2, 3 and 4 hrs were approximately 8.9%, 58.3%, 86.6% and 93% (Fig 6). Overall, melatonin showed significant protection of MT and CH quality at 1 hr of incubation with macrophage cells (p<0.05) compared to longer time periods. The control group had poor scores for approximately 20% of noncumulus and 16.6% for cumulus oocytes (p>0.05). Cumulus cells showed a non-significant protective effect against MPO secreted from stimulated macrophages in the presence and absence of melatonin at 2, 3, and 4 hrs of incubation (p>0.05). Collectively, the major cause for oocyte quality deterioration is the activation of macrophages as well as MPO that can be successfully inhibited by using melatonin.

Next, we co-cultured the oocytes with stimulated neutrophils as function of time, which yielded similar results. In the absence of cumulus cells, the poor scores for the oocytes incubated with stimulated neutrophils for 1, 2, 3 and 4 hrs were approximately 50%, 70%, 90% and 100% for MT and CH. In the presence of cumulus cells, the average poor scores for the oocytes incubated with stimulated macrophage cells for 1, 2, 3 and 4 hrs were approximately 50%, 65%, 80% and 100% for MT and CH. Overall, increasing the incubation time increased significantly the poor scores for MT and CH (p = 0.00) (data not shown). Cumulus cells showed a non-significant protective effect against MPO secreted from stimulated neutrophils at 2, 3, and 4 hrs of incubation (p = 0.9). Collectively, either utilizing stimulated macrophages or neutrophils, the major cause for oocyte quality deterioration is the activation MPO that can be successfully inhibited by using melatonin.

## Discussion

Recent studies have shown that intra-oocyte H_2_O_2_ concentration is relatively high and diffuses to the extracellular environment of the oocyte [31, 62]. Our current study confirms and extends these results and indicates that the diffused H_2_O_2_ deteriorates oocyte quality through MPO activation independent of cumulus cells and exogenously added H_2_O_2_, and could be prevented by treatment with melatonin, a potent inhibitor of MPO chlorinating activity [34–36]. Similarly, stimulated macrophages and neutrophils were also found to deteriorate oocyte quality independent of cumulus cells presence in a time dependent fashion, and could be prevented by melatonin. Macrophages and neutrophils are one of the principal defense mechanisms of innate immunity [63, 64] as a source of MPO and other toxic molecules used in controlled environments to degrade invading pathogens [24, 65]. Although, the association between macrophages and infertility has been repeatedly reported [66–68], the current work is the first to mechanistically link the MPO activity with the deterioration in the oocyte quality, which adversely influences infertility.

All the indications point to diffused intra-oocyte H_2_O_2_ being sufficient to trigger the MPO chlorinating activity (generation of HOCl), which was responsible for the loss of oocyte quality. Hydrogen peroxide is a naturally occurring molecule within the oocyte and a high portion appears to diffuse outside the oocyte [31]. Hydrogen peroxide is an uncharged stable molecule, and permits through biological membranes in a fashion similar to water [69, 70] via limited diffusion and transport through specialized proteins known as aquaporins [71]. MPO activated through intraoocyte diffused H_2_O_2_, was found to negatively affect oocyte quality in a time dependent manner in a similar fashion to that recently observed when oocytes were treated with increasing concentration of exogenous HOCl (Shaeib et al., in press. 2015). Treatment with HOCl disturbs the antioxidant capacity of cumulus cells by decreasing the number and/or viability of these protective cells (Shaeib et al., in press. 2015). Indeed, MPO treatment was found to mediate cumulus oocyte damage to almost the same extent as that in the absence of cumulus cells. HOCl may mediate oxidative damage and/or oocyte fragmentation through its ability to undergo numerous reactions with biomolecules, including aromatic chlorination, chloramine formation, aldehyde generation, and oxidation of thiols [4, 28]. Preservation of oocyte quality by melatonin provides further evidence for the involvement of MPO activation in causing oocyte quality deterioration [35, 36]. The ability of MPO to utilize melatonin as a one electron substrate to produce substances with less antioxidant potential, such as N1-acetyl-N2-formyl-5-methoxynuramine (AFMK) and N1-acetyl-5-methoxykynuramine (AMK), limits the duration of the oocyte protection by the amount of the melatonin provided [72, 73]. Previously, we have shown that pre-incubation of oocytes with increasing concentrations of melatonin prior to HOCl treatment significantly prevented HOCl-mediated deterioration of oocyte quality [36]. It has been further demonstrated that, under specific condition, melatonin treatment could significantly improve fertilization and pregnancy rates [74, 75]. This work provides a direct link between MPO and deterioration of oocyte quality leading to poor reproductive outcomes.

Several inflammatory diseases such as endometriosis, polycystic ovarian syndrome, diabetes, are not only associated with increased ROS production but also increased MPO levels [16–20, 76, 77]. In these disorders, elevated MPO levels have also been linked directly or indirectly with decline in fertility [2, 78]. We have previously showed that oocytes obtained from women with endometriosis display granulosa cells apoptosis, increased nitrotyrosine, premature cortical granule exocytosis in oocytes, disrupted microtubule morphology, and disrupted chromosomal alignment [79]. The deterioration of these oocyte quality parameters is not only caused by ROS but may also occur through MPO catalysis consistent with our current results [5, 31, 36]. Elevated MPO activity shifts the environment from one of host defense to one capable of host damage directly through the generation of ROS or indirectly through hemoprotein heme destruction and subsequent free iron release [29]. Free iron (most commonly Fe^2+^) through H_2_O_2_ driven Fenton reaction yields •OH, which propagates deterioration in oocyte quality contributing to the development of infertility [80–82]. MPO can also consume NO as a physiological one-electron (1e^-^) substrate [83]. Direct quantitative NO measurements utilizing NO-selective electrodes revealed that there is a significant amount of NO inside the oocyte [84]. NO deficiency has been shown to deteriorate oocyte quality and accelerate oocyte aging [50, 52]. Thus, MPO may damage the oocytes through multiple pathways: generation of oxidants such as HOCl and •OH, serving as a source of free iron, and depleting NO.

MPO is produced in high levels during inflammation throughout of the female reproductive tract from stimulated inflammatory cells such as neutrophils, monocytes, and macrophages [1, 85–87]. The distribution of macrophages in the ovary during different stages of oocyte development, as well as their presence in peri-ovulatory human follicular fluid, suggest that macrophages play important roles in folliculogenesis and tissue restructuring at ovulation [10, 11]. Indeed, during oocyte development in mice, rats, and humans, macrophages are recruited into the cellular layers of the follicle causing their numbers to be greatest just prior to ovulation [10, 12]. Activation of these macrophages or recruitment of other immune cells in the presence of inflammation for any reason can therefore contribute to deterioration in oocyte quality. Activated macrophages, like neutrophils, may mediate oocyte quality damage not only through triggering of MPO chlorinating activity, but also through ROS and cytokine cascades. Previously, we have shown that ROS such as O_2_^•-^, H_2_O_2_, ^•^OH, and ONOO^-^, as well as, IL-6, generated in the process of oxidative stress, not only regulates the inflammatory setting and contributions in keeping of chronic inflammatory state but also directly or indirectly affects the metaphase-II oocyte spindle and thus chromosomal alignment and significantly contributes to infertility [4, 5, 46, 51]. Similarly, treatment with melatonin (MPO inhibitor and ROS scavenger) highlights the culpability of immune cells in affecting oocyte quality.

Parallel to increased expression of MPO in inflammation, research has demonstrated elevations in macrophage concentration and activity in conditions such as polycystic ovarian syndrome and endometriosis related infertility [11, 88–91]. Non-activated peritoneal macrophages when co-cultured with the oocyte post fertilization leads to higher rates of development in in-vitro embryos when compared to the control group [56]. In support of the variable role of macrophages based on activation status, an association has been demonstrated between endometriosis and increased numbers of macrophages; however concluded based on colorimetric assay of MPO activity, that impaired function or abnormal activation, and not macrophages population size is important for endometriotic tissue proliferation [78, 92]. Similarly, high concentrations of inflammatory cytokines (TNF and IL-1) secreted from activated macrophages have been shown to cause deleterious effects on pre-implantation embryos [93]. Therefore, increased macrophage activation in the follicular fluid may cause a disruption in folliculogenesis and the deterioration in oocyte quality observed in pathologic conditions causing infertility [9, 66, 94, 95]. As shown in in vitro studies, oxidative states generated upon activating macrophages and neutrophils may also compromise oocyte quality by affecting the meiotic spindle and therefore the alignment of the chromosomes [4, 5, 31, 36, 46, 51, 61]. Thus, irrespective of whether purified MPO or activated macrophages are utilized, this works provides an initial mechanistic link between MPO activity and deterioration in oocyte quality.

In addition to its ability to serve as a potent inhibitor of the chlorinating activity of mammalian peroxidases and a scavenger of ROS, melatonin serves as a transition metal chelator, thereby reducing the downstream adverse effects such as lipid peroxidation, protein oxidation, and DNA damage [36, 96–99]. Melatonin is also unique among other HOCl scavengers, as its oxidation products have no biologically deleterious effects and therefore has broad therapeutic indications including cardiovascular disease, immune dysfunction, sleep disturbance and subfertility [100, 101]. Collectively, melatonin’s ability to inhibit the chlorinating activity of MPO or scavenging neutrophil or macrophage driven HOCl might be a useful therapeutic approach in reducing adverse reproductive outcomes caused by inflammation mediated deterioration in oocyte quality.

In conclusion, our current work showed for the first time the link between stimulated- macrophages and neutrophils, major sources of MPO, and oocyte quality deterioration, highlighting the implications of these cells in infertility caused by inflammatory conditions. Melatonin has potential therapeutic effects in preserving oocyte quality, thus improving reproductive outcomes in patients with chronic inflammation.
